# Innate Lymphoid Cells and Intestinal Inflammatory Disorders

**DOI:** 10.3390/ijms23031856

**Published:** 2022-02-06

**Authors:** Mingzhu Zheng, Jinfang Zhu

**Affiliations:** 1 Molecular and Cellular Immunoregulation Section, Laboratory of Immune System Biology, National Institute of Allergy and Infectious Diseases, National Institutes of Health, Bethesda, MD 20892, USA; 2 Jiangsu Provincial Key Laboratory of Critical Care Medicine, Department of Microbiology and Immunology, Southeast University, Nanjing 210009, China

**Keywords:** ILC1, ILC2, ILC3, intestinal inflammation

## Abstract

Innate lymphoid cells (ILCs) are a population of lymphoid cells that do not express T cell or B cell antigen-specific receptors. They are largely tissue-resident and enriched at mucosal sites to play a protective role against pathogens. ILCs mimic the functions of CD4 T helper (Th) subsets. Type 1 innate lymphoid cells (ILC1s) are defined by the expression of signature cytokine IFN-γ and the master transcription factor T-bet, involving in the type 1 immune response; ILC2s are characterized by the expression of signature cytokine IL-5/IL-13 and the master transcription factor GATA3, participating in the type 2 immune response; ILC3s are RORγt-expressing cells and are capable of producing IL-22 and IL-17 to maintain intestinal homeostasis. The discovery and investigation of ILCs over the past decades extends our knowledge beyond classical adaptive and innate immunology. In this review, we will focus on the roles of ILCs in intestinal inflammation and related disorders.

## 1. Introduction

The immune system is the most important part of body defense system against viruses, bacteria, and fungi. It is composed of specialized innate and adaptive immune cells. T cells and B cells are the adaptive immune cells, whereas innate lymphoid cells (ILCs) are the innate counterparts of CD4^+^ T helper (Th) cells based on the similarity in their development and functions. ILCs lack T cell antigen receptors, B cell receptors, and other myeloid cell markers, and are thus defined as lineage negative (Lin^−^) cells. However, ILCs express IL-7 receptor α chain (CD127 or IL-7Rα) with some of them also expressing IL-2 receptor α subunit (CD25). ILCs can mediate protective and repair functions through cytokine secretion.

**Figure 1:**
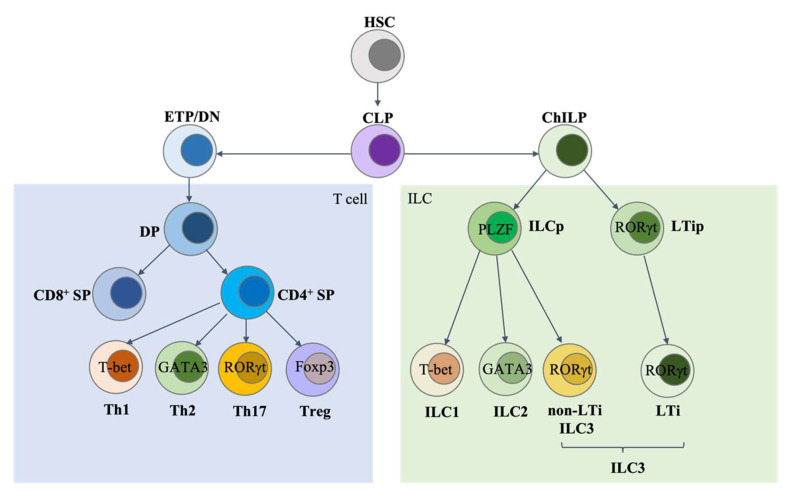
ILC development. There are several similarities between the development of innate lymphoid cells (ILCs) and T cells. Both T cells and ILCs are derived from common lymphoid progenitors (CLPs). T cells go through the CD4/CD8 double-negative (DN) and double-positive (DP) stages and then differentiate into CD4 or CD8 single positive (SP) cells. CD4 SP cells can further differentiate into Th1, Th2, Th17, or Tregs in certain environments. All ILCs are generated from common helper-like innate lymphoid progenitors (ChILPs). While the non-LTi ILCs are generated from PLZF-expressing common progenitors (ILCPs), LTi cells develop from a distinct progenitor which has no history of PLZF expression but depends on the transcription factor RORγt for their development. As during T helper (Th) cell differentiation, ILCPs can give rise to ILC1, ILC2, and ILC3 subtypes, whose development depends on the master transcription factors T-bet, GATA3, and RORγt, respectively. ETP, early thymic progenitors. Background color: blue: T cell lineage; green: ILC lineage.

T cells originate from common lymphoid progenitors (CLPs) in the bone marrow and develop in the thymus. ILCs are also derived from CLPs in both fetal liver and adult bone marrow. For ILC development, CLPs first differentiate into common helper-like innate lymphoid progenitors (ChILPs), which are Id2-expressing ILC progenitors [1]. Then, ChILPs can further develop into innate lymphoid common progenitors (ILCPs) that transiently express transcription factor PLZF, and the ILCPs have a limited capacity to become conventional natural killer (cNK) cells. ILCPs can give rise to all ILCs except for lymphoid tissue inducer (LTi) cells, and LTi cells are derived from LTi progenitors without a history of PLZF expression (Figure 1). Our recent work revealed an import role of the expression level of the transcription factor GATA3 in determining LTi and non-LTi ILC lineage fate [2].

**Figure 2:**
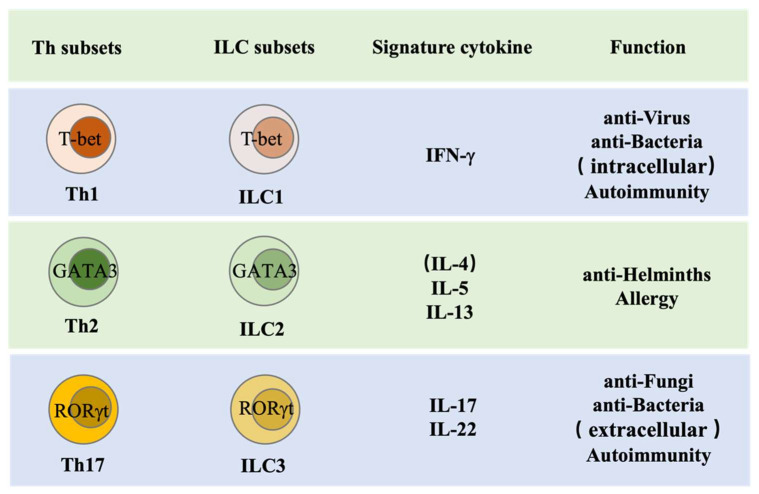
ILC function. At the maturation stage, CD4^+^ Th cells and ILC subsets produce similar effector cytokines and share same master transcription factors for their functions. Th1 cells and ILC1s are capable of producing IFN-γ and are important for the clearance of viruses, intracellular bacteria, or protozoa and mediate autoimmunity. Th2 cells and ILC2s, by producing IL-5 and IL-13, are important for helminth expulsion and are involved in allergy. Th17 cells and ILC3s, through secreting IL-17 and IL-22, are critical for protective immune responses against fungal and extracellular bacterial infections and contribute to autoimmunity.

Based on the master transcription factor expression and signature cytokine secretion, ILCs are divided into three major groups, namely ILC1s, ILC2s, and ILC3s, which are the innate counterparts of Th1, Th2, and Th17, respectively. ILCs may respond earlier and more quickly than T helper cells during infection, as they reside and can respond to inflammatory cytokines in the tissue [3] (Figure 2). ILCs may serve as a bridge between the innate and adaptive immune system. Innate cells in the myeloid compartment can directly sense pathogen invasion by producing inflammatory cytokines to activate ILCs and by presenting antigen to adaptive cells through antigen-specific recognition. While ILCs can directly secrete effector cytokines to orchestrate local immune response, they are also capable of presenting antigens to Th cells through MHCII and thus promoting T helper (Th) cell differentiation and effector functions [4,5,6,7].

The intestinal immune system represents the largest compartment of the immune system, as the intestinal tract is the largest internal extension of the body’s surface [8,9,10]. The intestine is constantly challenged by a huge number of stimuli including microbes and food-derived antigens. A number of immune cells within the distinct types of lymphoid structures are thought to maintain the homeostasis and functions of the gut immune system. These gut-specific lymphoid structures including Peyer’s patches (PPs) and isolated lymphoid follicles (ILFs) are located in the lamina propria (LP) [8,11,12,13,14,15]. Both genetic programs and environmental inputs such as luminal microbial stimulation promote the development of ILFs postnatally, whereas PPs are only dependent on the genetic program in the fetal stage. ILFs are composed mainly of B cells surrounded by LTi-like ILC3s [16,17]. Several B cell follicles and distinct T and B cell areas are present in PPs, which represent the most structured lymphoid organs in the intestine [11]. ILCs, mainly ILC3s, are located at the small intestine lamina propria (siLP). They are the first line in the gut to fight against the pathogens; therefore, their dysfunction will result in intestinal disorders such as inflammation. In this review, we will focus on the major subsets of ILCs and their functions in the intestine, in the context of pathogenic microbe-induced inflammatory disorders.

## 2. ILC1s and Related Intestinal Inflammatory Disorders

### 2.1. ILC1s

Group 1 innate lymphoid cells (ILC1s) are believed to express T-box transcription factor T-bet and produce high levels of IFN-γ, and thus protect the host against infections with certain viruses, bacteria, and protozoa, such as the intracellular parasite *Toxoplasma gondii* [1]. ILC1s express several natural killer (NK) cell markers, such as NK1.1 and NKp46 [18,19]. However, they are distinct from NK cells, as they lack the expression of perforin, granzyme B as well as the NK cell markers CD56, CD16, and CD94, which result in less cytotoxicity than NK cells [20]. Many ILC1s express CD127 (IL-7Rα) and depend on IL-7 and IL-15 signaling for their differentiation and/or survival [21]. Some “ILC1s” could also develop from RORγt^+^ ILC3s under the influence of IL-12 [20]. ILC1s preferentially express CD49a, TRAIL, and CD103, which are tissue-dependent; however, the expression of some of these markers may get lost upon ILC1 activation [18,22,23]. Unlike circulating NK cells, ILC1s are largely tissue-resident; ILC1s from intestine and liver are CD49a^+^CD49b^−^ and do not express the transcription factor Eomes [1].

### 2.2. ILC1-Related Intestinal Inflammatory Disorders

ILC1s are the first line of defense against many infections. They release high amounts of IFN-γ in response to IL-12 and IL-15 stimulation and are critical for host defense during intestinal infections [24,25,26]. In humans, ILC1s are enriched in the upper gastrointestinal (GI) tract [27].

By using a T-bet-deficient mouse model, scientists have revealed critical functions of ILC1s during infection. ILC1s are the major producers of IFN-γ and TNF-α in the gut during *Toxoplasma gondii* infection, and T-bet-deficient mice that lack ILC1s fail to control parasite replication during the early stage of infection [1]. T-bet deficiency within the innate immune compartment results in spontaneous colitis triggered by *Helicobacter typhlonius*, which drives excess TNF-α production and promotes colitis in *Tbx21*^−/−^*Rag2*^−/−^ ulcerative colitis (TRUC) mice [28]. ILC1s are also involved in protection against intracellular *Salmonella enterica* infection. During *Salmonella* infection, T-bet-expressing ILCs are the main source of IFN-γ, which promotes the secretion of glycoproteins that are essential to form mucus and protect the epithelial barrier [29]. Similarly, Abt et al. demonstrated that the absence of ILCs lead to increased susceptibility to *Clostridium difficile*, and *Rag2*^−/−^*Il2rg* ^−/−^ mice were protected by adoptively transferred ILCs during acute *Clostridium difficile* infection. In this infection model, ILC1s but not ILC3s make a major contribution to host resistance; loss of IFN-γ or T-bet-expressing ILC1s in *Rag1*^−/−^ mice are susceptible to *C. difficile* infection, while loss of ILC3s or IL-22 production has a limited impact on the recovery following *C. difficile* infection [30]. In addition, Schroeder et al. reported that continuous expression of T-bet in mature cells is critical for the maintenance of ILC1s, as evidenced by induced deletion of T-bet in a Rosa26-Cre-ERT2 model that resulted in the absent of intestinal ILC1s. In contrast, neither colonic lamina propria (cLP) ILC2s nor ILC3s were altered upon T-bet deletion. Although STAT1 and STAT4 are important for inducing T-bet in T cells, they are not required for the development and maintenance of intestinal ILC1s. Induced deletion of T-bet in mice results in protection from the severe colitis in vivo, suggesting a critical role of ILC1s in this disease [31].

c-FLIP is an inhibitory protein crucial for T cell development and is upregulated during the activation of both human and mouse T cells. Bank et al. discovered c-FLIP expression on the NKp46^+^ ILCs were significantly upregulated in response to IL-15. By crossing NKp46^iCre^-transgenic mice with c-FLIP^fl/fl^ mice, they further showed that NKp46^+^ ILC-specific deletion of c-FLIP led to the loss of all the IL-7/IL-15-dependent NKp46^+^ ILCs, subsequently inducing early-onset chronic colitis and microbial dysbiosis [32]. Stimulator of interferon genes (STING) is a cytosolic sensor that can interact with cyclic dinucleotides (CDNs) and intracellular DNA [33,34]. STING plays a central role in the host defense against pathogens and the development of autoimmune diseases through its abnormal activation by self-DNA [35,36]. The role of STING in regulating intestinal tumorigenesis in mice was underscored recently [37,38]. Canesso et al. demonstrated that STING is vital for controlling intestinal inflammation. STING-deficient mice are highly susceptible to dextran sodium sulfate (DSS)-induced colitis, T cell-induced colitis, and enteric *Salmonella typhimurium* infection compared with wild type (WT) animals. Inactivation of STING leads to a reduction in intraepithelial lymphocytes (IELs) and ILC2s, but an increase in ILC1s and ILC3s in the colon. Therefore, ILC1s and ILC3s play a role in maintaining gut homeostasis and a protective effect in controlling gut inflammation [39].

Under other circumstances, for example, the commensal bacteria translocate from the gut lumen into the lamina propria when the gut barrier is damaged, ILC1s are the major player in facilitating the intestinal anti-bacterial defense through the production of IFN-γ. By using an in vitro human colonic lamina propria mononuclear cell (LPMC) model, Castleman et al. demonstrated that only gram-negative commensal or pathogenic bacteria induce significant IFN-γ production. ILC1s respond to IL-12, IL-18, and IL-1β produced by myeloid cells after the recognition of gram-negative bacteria [40].

The frequency of ILC1s is higher in inflamed intestines of patients with Crohn’s disease (CD), indicating an important role of these IFN-γ-producing ILC1s in the pathogenesis of intestinal mucosal inflammation [20,41]. Furthermore, by using gut organoid cocultures with ILC1s, Geraldine et al. elucidated a previously undescribed interaction between ILC1s and their microenvironment and demonstrated that murine and human ILC1s promote intestinal epithelial and matrix remodeling through secreting transforming growth factor β1 (TGF-β1) [42].

## 3. ILC2s and Related Intestinal Inflammatory Disorders

### 3.1. ILC2s

Group 2 innate lymphoid cells (ILC2s) confer protective type 2 immunity during helminth infection and are also involved in allergic inflammation or tissue repair. ILC2s express high levels of GATA3 and secrete IL-5 and IL-13 upon stimulation. ILC2s respond to the cytokines IL-25, TSLP, and IL-33. The immature ILC2 progenitors in the bone marrow express CD25, Sca-1, and T1/ST2; however, only the T1/ST2 positive population does not include potential progenitors that express low levels of GATA3. Thus, T1/ST2 is a better marker than CD25 or Sca-1 to distinguish immature ILC2s from the ILC progenitors [2]. ILC2s can also trans-differentiate into ILC1s after IL-12/18 stimulation [43,44].

We and others have previously demonstrated that GATA3 is essential for ILC2 development and maintenance [2,45,46,47,48]. T cell factor 1 (TCF-1, encoded by *Tcf7* gene), another important transcription factor for T cell lineage commitment, has also been implicated in the development of ILC2s [49]. TCF-1 functions through both GATA3-dependent and GATA3-independent pathways to facilitate ILC2 generation. TCF-1 is intrinsically required for the development of both ILC2s and NKp46^+^ ILC3s [50]. Bcl11b, a third critical transcription factor for T cell development, sustains ILC2 lineage commitment and functions [51,52] by directly regulating the expression of an ILC2-related transcription factor Gfi-1; Bcl11b-deficient ILC2s express lower levels of GATA3 and its downstream genes, including IL-33 receptor (encoded by the *Il1rl1* gene) but aberrantly express RORγt and other ILC3-related genes. The development of ILC2s, but not other ILCs, is also dependent on the transcription factor retinoic acid receptor-related orphan receptor (RORα), although it is expressed in the common ILC progenitors [53].

### 3.2. ILC2-Related Intestinal Inflammatory Disorders

Like ILC1s, ILC2s are also largely tissue-resident, and they maintain and may expand locally under physiologic conditions [54,55]. In addition to lung and skin tissues, ILC2s are also located at the intestinal lamina propria and fat-associated lymphoid clusters of the intestinal mesentery. Vitamin A deprivation will result in diminished ILC3s and in turn contribute to impaired immunity to acute bacterial infection. In contrast, ILC2s will expand with vitamin A deficiency, which contributes to enhanced efficiency for parasitic helminth expulsion [7]. Huang et al. reported the existence of a subpopulation of ILC2s, namely inflammatory ILC2 (iILC2). These iILC2s are responsive to interleukin 25 (IL-25), whereas natural ILC2 (nILC2) cells are IL-33-responsive. In that study, it was shown that iILC2s are circulating cells originated from resting ILC2s in intestinal lamina propria. Through sphingosine 1-phosphate (S1P)-mediated chemotaxis, iILC2s migrate to various tissues, including lung, to contribute to anti-helminthic immune response and tissue repair [56,57,58]. Notch signaling plays an important role in inducing ILC2 plasticity by driving the conversion of nILC2s into highly proinflammatory iILC2s. Acute blockade of Notch signaling abolishes functional iILC2s, but not nILC2s [59]. Interestingly, these iILC2s can develop into nILC2-like cells in vitro and in vivo and contribute to the expulsion of *Nippostrongylus brasiliensis*. They may also acquire the capacity of producing IL-17 and provide partial protection against *Candida albicans* [56,57,58]. The frequency and total cell numbers of intestinal ILC2s are identical in specific pathogen free (SPF) mice compared to germ free (GF) mice; however, the gene expression profile of ILC2s can be influenced by the microbiota [60,61]. ILC2s are capable of secreting amphiregulin (AREG) to protect intestinal damage and inflammation in acute DSS colitis [62]. In addition, ILC2s produce large amounts of IL-13 in the lamina propria in oxazolone colitis [63]. IBD-related protein signaling lymphocyte activation molecule family member 1 (SLAMF1) is found upregulated on ILC2s from both CD and ulcerative colitis (UC) patients compared to healthy controls. In line with the systemic nature of CD inflammation, the finding that circulating SLAMF1 positive ILC2s are increased in patients with inactive disease suggests that the expansion of SLAMF1 positive ILC2s may be considered as an interesting therapeutic strategy [64].

Loss of TCF-1 expression impairs the capacity of ILC2s to produce IL-5 and IL-13 and results in crippled responses to intestinal infection with *Citrobacter rodentium* [50]. Core binding factor β (CBFβ) is also required for ILC2 activation. Upon CBFβ deletion, the maintenance of ILC2s in steady state is not impaired, but ILC2s are inactivated during allergic airway inflammation. CBF transcriptional complex directly binds to the promoters and enhancers of the *Il13* and *Vegfa* gene loci and controls their gene transcription [65]. Dedicator of cytokinesis 8 (DOCK8) is a guanine nucleotide exchange factor (GEF) for Cdc42. Deletion of DOCK8 induces a skewing to type 2 immunity in the gut by promoting ILC2 expansion, indicating that DOCK8 is a negative regulator of intestinal ILC2s via Cdc42 activation [66].

Gut ILC2 activation can be regulated by the nervous system, as evidenced by the fact that ILC2s can be activated through its Nmur1 receptor via binding with the ligand neuromedin U (NMU) released from neurons, thereby supporting efficient *Nippostrongylus brasiliensis* expulsion [67,68]. By contrast, another neuropeptide, named alpha-calca-encoding calcitonin gene-related peptide (a-CGRP), is predominantly expressed in vivo in choline acetyltransferase (ChAT^+^) enteric neurons [69], and CGRP limits ILC2 responses and constrains type 2 inflammation. Without CGRP signaling, ILC2-mediated responses and worm expulsion are enhanced [70]. Thus, the cross-talk between the enteric nervous system and ILC2s is critical for establishing intestinal immune homeostasis. Similarly, PD-1 is found to be an important negative regulator of KLRG1 positive ILC2 functions in both mice and humans. During *Nippostrongylus brasiliensis* infection, a significantly higher proliferation of KLRG1 positive ILC2s are found in *Pdcd1*^−/−^ mice compared to WT mice. Adoptive transfer of *Pdcd1*^−/−^ KLRG1^+^ ILC2s into *Rag2*^−/−^ *γc*^−/−^ mice can dramatically reduce worm burden compared to WT KLRG1^+^ ILC2 reconstituted mice. Furthermore, increased KLRG1^+^ ILC2s resulting from blocking PD-1 signaling will reduce disease severity. Therefore, PD-1 is required for limiting KLRG1^+^ ILC2 cell number and functions [71].

ILC2s interact with Th2 cells via MHCII to promote parasitic helminth expulsion. McKenzie and colleagues use two different ILC2 cell-deficient mouse strains to show that ILC2s acquire and process antigens and can induce activation and proliferation of antigen-specific T cells with an efficiency lower than that of CD11c^+^ dendritic cells (DCs) but equivalent to that of plasmacytoid dendritic cells (pDCs) or naive B cells. These MHCII-positive ILC2s interact with antigen-specific T cells, which produce IL-2 to promote ILC2 proliferation and IL-13 production. Deletion of MHCII resulted in a reduced capacity of IL-13-expressing ILC2s in efficiently expelling *Nippostrongylus brasiliensis*. Thus, during innate immunity transition to adaptive T cell-mediated immunity, the crosstalk between ILC2s and T cells contributes to their mutual maintenance, expansion, and cytokine production [72].

## 4. ILC3s and Related Intestinal Inflammatory Disorders

### 4.1. ILC3s

Type 3 innate lymphoid cells (ILC3s) are the most abundant ILCs in the gut and are involved in the innate immune responses against bacterial infection and maintenance of gut microbiota composition [73,74,75,76,77,78,79,80]. ILC3s express the transcription factor RORγt and depend on IL-7 for their development [81]. Some ILC3s could up-regulate T-bet to suppress RORγt expression and become IFN-γ-expressing cells when exposed to IL-12/18 [20]. ILC3s maintain intestinal homeostasis by producing IL-22 and IL-17 [82,83,84]. There are two subsets of ILC3s based on differential expression of cell surface markers CCR6 and NCR—one is CCR6^+^ LTi and LTi-like cells, and the other is NCR^+^ ILC3s. LTi cells, recognized long before the establishment of the ILC concept, play a critical role in lymphoid organogenesis [85]. LTi cells develop and function mainly at the fetal stage, whereas LTi-like cells are generated during adulthood. LTi-like cells can also produce the ILC3 signature cytokine IL-22 and play a protective role against pathogens [84,85]. NCR^+^ ILC3s are NKp46^+^ in mice and NKp44^+^ in human. NCR^+^ ILC3s can produce IL-22 and IL-17 as well as GM-CSF [86]. In mice, NKp46^+^ ILC3s are T-bet dependent and can also produce IFN-γ [29,87,88].

### 4.2. ILC3-Related Intestinal Inflammatory Disorders

ILC3s are enriched in the ileum [20,89] and colon [27]. In the steady state, the major function of ILC3s is to maintain the homeostasis of the intestinal barrier and to keep the balance between gut microbiota and immune cells. Once they sense the bacterial antigen, DCs and mononuclear cells will produce a large amount of IL-23 and IL-1β, which in turn stimulate ILC3s to produce the effector cytokines IL-22, IL-17, and GM-CSF. The aryl hydrocarbon receptor (Ahr) controls the development of adult but not fetal RORγt^+^ ILC3s in mice. Ahr is also critical for IL-22 expression, since Ahr-deficient ILC3s produce less IL-22 [90,91,92]. Similarly, dietary vitamin A deficiency results in abnormal ILC3s, reduced IL-22 production, and susceptibility of mice to *Citrobacter rodentium* infection [7]. Furthermore, RORγt^+^NKp46^+^ IL-22-producing ILCs contribute to host defense during intestinal damage in murine colitis models. These cells are localized in the intestine in normal and DSS-induced colitis in the *Rag2*^−/−^ mice. Interestingly, IFN-γ-producing ILC1s are decreased in RORγt-deficient *Rag2*^−/−^ mice, which develop more severe colitis induced by DSS, accompanied with lower expression of REG3b and REG3c in the colon [93].

T-bet controls ILC3 cellularity but does do not drive a pathogenic role of ILC3s in mice with a conventional SPF microbiota. T-bet-deficient mice have an increase in NKp46^−^ ILC3s accompanied with enhanced expression of RORγt and IL-7R, but independent of STAT1 or STAT4 signaling pathways. However, T-bet-deficient mice do not have a greater risk to develop spontaneous colitis [94]. A catalytic subunit of the mammalian chromatin remodeling BAF complex Brg1 (encoded by brahma-related gene 1) is known to regulate the development and function of various immune cells. When Brg1 is specifically deleted in ILC3s, the conversion of NKp46^−^ ILC3s to NKp46^+^ ILC3s was blocked because of the failure of T-bet upregulation in NKp46^−^ ILC3s [95]. Strikingly, *Brg1*^−/−^*Rag1*^−/−^ ILC3s produce increased amounts of GM-CSF and develop spontaneous colitis [95].

Epithelial fucose is used as a dietary carbohydrate by many commensal bacteria, which can induce epithelial fucosylation. Goto et al. report that ILC3s induce intestinal epithelial fucosyltransferase 2 (Fut2) expression and subsequent fucosylation in mice. This induction requires the IL-22 and lymphotoxin in a commensal bacteria-dependent and -independent manner, respectively. Disruption of intestinal fucosylation results in increased susceptibility to infection by *Salmonella typhimurium* [96].

The activation of ILC3s has rhythm. Wang et al. report that the clock regulator REV-ERBα (encoded by *Nr1d1*) has the prominent and rhythmic expression in ILC3s, which is associated with rhythmic cytokine expression [97]. Development and functions of the NKp46^+^ ILC3 subset is markedly impaired in REV-ERBα-deficient mice, as evidenced by reduced cell number, RORγt expression, and IL-22 production. REV-ERBα also has circadian-independent impacts on ILC3 development and functions through the regulation of RORγt [97]. VIP receptor type 2 (VIPR2; also known as VPAC2) selectively expressed on ILC3s can be activated by vasoactive intestinal peptide (VIP) expressed on enteric neurons; the production of VIP triggered by food intake can limit the expression of IL-22 by ILC3s. It has also been reported that the neuroimmune circuit in the intestine dynamic promotes IL-22-mediated innate immune protection [98]. In contrast, another group has demonstrated that VIP interacts with VIPR2 expressed on intestinal ILC3s, which results in increased production of IL-22 and enhanced barrier function of the epithelium. Conversely, deficiency of VIP-VIPR2 signaling results in impaired production of IL-22 by ILC3s and animals’ susceptibility to inflammation-induced gut injury [99].

However, ILC3s function as a double-edged sword in intestinal inflammatory diseases. In the physiologic state, ILC3s maintain gut microenvironmental homeostasis through the production of IL-22, IL-17, and GM-CSF at reasonable amounts to protect gut epithelia from microbe invasion. On the other hand, in the pathological state, ILC3s may transit towards an ILC1 phenotype. These dysfunctional ILC3s may over-produce IL-22/IL-17 and IFN-γ, which leads to the progression and aggravation of IBD [100]. ILC3s also participate in intestinal allografts. ILC1s and ILC3s are present in the epithelial compartment of functional human intestinal allografts. The balance among proinflammatory and homeostatic roles of ILC subsets will determine the viability of intestinal grafts [101].

ILC3s communicate with other immune cells such as Th cell subsets in the gut. Firstly, ILC3s could negatively regulate Th17 cells. Commensal segmented filamentous bacteria (SFB) will expand in Ahr-deficient mice due to reduced IL-22, and these mice have enhanced Th17 cell differentiation. *Rorc*^gfp/+^*Ahr*^−/−^ mice have a more severe reduction of ILC3s compared to *Rorc*^+/+^*Ahr*^−/−^ mice and are prone to spontaneous colitis. Thus, Ahr-expressing ILCs can limit T cell-mediated experimental colitis by suppressing pathogenic Th17 cells. These data indicate an intricate balance between ILC3s and Th17 cells regulated by Ahr and microbiota [102]. Secondly, ILC3s can support Treg cell survival and/or expansion in the intestine through IL-2 to prevent chronic gut inflammation Notably, T cell-specific deletions of IL-2 do not affect Tregs in the small intestine. Unexpectedly, the dominant source of IL-2 is ILC3s, and IL-2 is induced selectively by IL-1 β. Studies show that ILC3-derived IL-2 is required for Treg maintenance and oral tolerance to dietary antigens in the gastrointestinal tract. Furthermore, Crohn’s disease patients produce less IL-2 by ILC3s in the small intestine, and this is also relevant to lower frequencies of Tregs. Thus, the protective effects of IL-2 contain the generation, maintenance, and function of Tregs, and the potential therapeutic strategy for patients with IBD is the usage of low doses of IL-2 [103]. Thirdly, Mao et al. identified that CD4^+^ T cells can also regulate ILC3 activation in the intestine. In WT mice, phosphorylated-STAT3 is only transiently induced by microbial colonization at the weaning stage, when CD4^+^ T cell-mediated immunity is still being developed to control the expanding commensal burden. By contrast, the persistent phosphorylation of STAT3 in ILC3s induced by IL-23 was observed in the absence of CD4^+^ T cells. As a result, the persistent IL-22 production from ILC3s in T cell-deficient mice results in impaired host lipid metabolism in the small intestine. These findings provide new insights into how innate and adaptive lymphocytes synthesize to establish steady-state commensalism and metabolic tissue homeostasis in distinct ways during normal development [104].

## 5. Concluding Remarks

**Figure 3:**
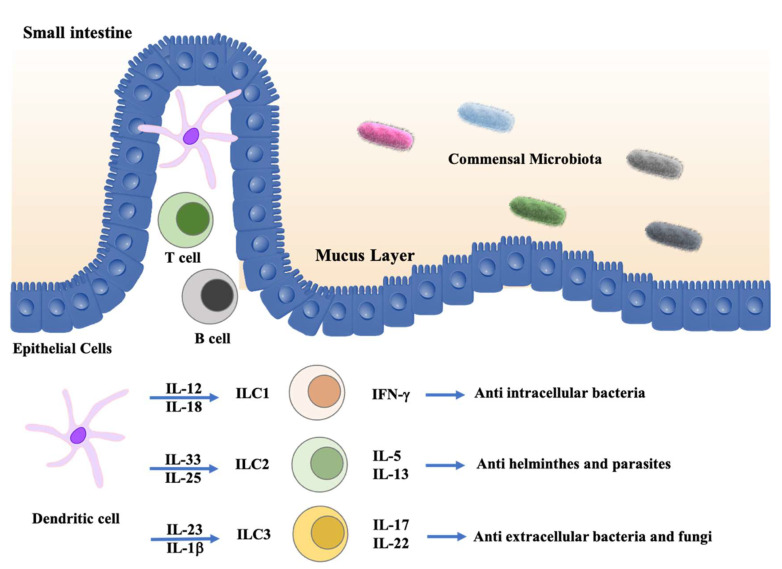
ILCs in intestine. In the healthy intestine, ILCs maintain intestinal homeostasis through induction of protective immune responses against pathogenic microbes and keep the intestinal barrier integrity. In response to microbial stimuli, dendritic cells (DCs) and macrophages may stimulate ILC1 responses to intracellular pathogens through secreting IL-12 and IL-18, and they induce ILC3 responses against extracellular bacteria and fungi through secreting IL-23 and IL-1β. On the other hand, epithelial-derived factors, such as IL-33 and IL-25, may induce type 2 cytokine secretion by ILC2s and in turn contribute to the expulsion of helminths.

Over 10 years of intensive research, distinct ILC subsets and their functions have been well studied (Figure 3). ILCs can respond earlier and more quickly than adaptive immune cells against the invasion of bacteria, helminths, and fungi. The intestine is the largest mucosal site, where ILCs reside and microbiota are critical in modulating intestinal homeostasis, partly through their interactions with ILCs [105,106,107].

Especially for ILC3s, they seem to be the major ILC subset involved in intestinal homeostasis through IL-22 and IL-17 production to protect the host from pathogen invasion. As mentioned above, vitamin A is important for ILC3s to fight against intestinal *Citrobacter rodentium* infection. In addition, intestinal fucosylation impairment will result in increased susceptibility to *Salmonella typhimurium* infection. Furthermore, the reduction of either ILC3 master transcription factor expression or signature cytokine production will contribute to intestinal inflammation disorders. For example, Ahr is a critical regulator for adult ILC3 development and IL-22 production, and therefore, the Ahr-deficient mice are more prone to infections. While LTi cells can also participate in host defense, they have a unique function in organogenesis of lymphoid structures.

ILC1s, by producing IFN-γ and TNFα, may be involved in host defense against infections with viruses, bacteria, and protozoa including *Toxoplasma gondii*. In addition, loss of IFN-γ or T-bet-expressing ILC1s in *Rag1*^−/−^ mice increases susceptibility to *C. difficile*. Furthermore, STING-deficient mice are more prone to DSS-induced colitis and enteric *Salmonella typhimurium* infections. Anti-helminth host defense and tissue repair are the key functions of ILC2s. Notch signaling, NUM, as well as MHCII are essential for ILC2s to fight against *Nippostrongylus brasiliensis* infection.

Regulatory ILCs (ILCregs) were initially reported by Zusen Fan’s lab in 2017. In their work, ILCregs were found in both mouse and human intestines. They also showed that ILCregs can suppress the activation of ILC1s and ILC3s via secretion of IL-10 to control the innate intestinal inflammation [108]. However, scientists in Marco Colonna’s lab were not able to detect these IL-10-producing ILCregs in a variety of mouse strains. Instead, they found that the major source of IL-10 is ILC2s that have been activated. IL-2, IL-4, IL-27, IL-10, and NMU can enhance IL-10 production by ILC2s. Whether IL-10 can be produced by other known or unknown ILC subsets in mice and in human requires further investigation [109].

All the ILC subsets can be involved in different forms of intestinal inflammatory disorders; however, their precise roles in immunity and inflammation remain to be carefully addressed because of the redundant functions of T cells. More specifically, the roles of gut-resident ILCs shaped by the gut environment, which includes the interaction network between ILCs, other intestinal cells and microbiota, need further exploration. In addition, the roles of ILCs in the onset and maintenance of gut inflammation, and the expression of the surface checkpoints, should encourage the researchers to explore innovative therapeutic targets that regulate the activation and functions of ILCs in treating intestinal inflammatory diseases.
